# A prospective evaluation of tibial insertion sites for intraosseous needles to gain vascular access in Asian neonates

**DOI:** 10.1038/s41372-024-02018-x

**Published:** 2024-06-06

**Authors:** Chutima Sengasai, Preeyacha Pacharn, Bosco Paes, Ratchada Kitsommart

**Affiliations:** 1 Division of Neonatology, Department of Pediatrics, Faculty of Medicine Siriraj Hospital, Mahidol University, Bangkok, Thailand; 2 Division of Diagnostic Radiology, Department of Radiology, Faculty of Medicine Siriraj Hospital, Mahidol University, Bangkok, Thailand; 3 Division of Neonatology, Department of Pediatrics, McMaster University, Hamilton, ON Canada

**Keywords:** Outcomes research, Paediatrics

## Abstract

**Objectives:**

To determine the appropriate intraosseous (IO) needle insertion site, optimal depth and success using a drill-assisted device (DAD) versus a manually inserted needle (MIN).

**Methods:**

Computed tomography scans of neonatal cadavers were analyzed. Success was based on tibial needle tip placement within the marrow cavity and contrast media distribution.

**Results:**

Nineteen cadavers (38 tibiae) were included. The overall success rate was comparable between DAD and MIN needles, but reduced in very-low birthweight (VLBW) infants. The insertion site was consistent across birth weight groups. Contrast leakage occurred overall in 15.8% and 41.7% in VLBW infants and was insignificantly greater in DAD versus MIN needles. Minimum and maximum puncture depth was adjusted for higher BW groups.

**Conclusion:**

IO needles should be placed 2 cm below and 1–2 cm medial to the tibial tuberosity. MIN needles are preferred to minimize leakage. IO depth should be modified by birth weight.

## Introduction

Neonatal cardiopulmonary resuscitation entails a sequential approach based on the severity of the infant’s condition and their responsiveness. Although ventilation is considered paramount in the resuscitation steps, approximately 0.5% of infants need epinephrine to enhance perfusion of the coronary arteries and oxygen transport [1, 2]. When indicated, epinephrine and fluid volumes should swiftly enter the central circulation through umbilical venous lines. If venous access cannot be achieved, epinephrine can be administered via either the intratracheal or intraosseous (IO) route [3]. The IO route, however, affords sole access for delivering both epinephrine and fluid boluses. This involves needle access to the marrow space in flat bones, usually the tibia, whereby medications and fluids rapidly enter the central circulation, akin to venous delivery [4].

In term neonates, the Neonatal Resuscitation Program (NRP) 8th edition recommends IO insertion into the flat, lower leg bones, approximately 2 cm distal and 1–2 cm medial to the tibial tuberosity [3]. A distinct change of resistance indicates needle penetration into the medulla. In a sub-Saharan African neonatal cadaver study, a distance of approximately 10 mm below the tibial tuberosity was recommended for IO insertion [5]. Procedural complications include extravasation that could impact resuscitation efficacy and possibly result in organ loss or bone fractures in 15–30% [6, 7]. The NRP guideline lacks clear recommendations regarding needle depth for both term and preterm neonates and does not account for ethnic differences affecting neonatal weight [8]. Variations in long bone length and proper needle insertion sites may differ in Asian versus American and African neonates because of their smaller size [8].

IO insertion in neonates can also be challenging due to thinner bones and difficulty sensing change of resistance, which can impact accurate penetration depth [6]. Inappropriate IO insertion sites may lead to severe complications, such as infection, bone fractures, and limb ischemia [6]. Moreover, data are lacking on success rates of IO needle insertion at recommended sites, especially in Asian neonates, including preterms. The information is necessary to determine if the general NRP guidelines on IO insertion pertain equally to Asian neonates. We anticipated that the recommended IO needle insertion site outlined in the NRP 8th edition guideline in the proximal tibia would be applicable in Asian neonates.

Therefore, our primary objective was to investigate whether the recommended IO needle insertion site at approximately 2 cm distal and 1–2 cm medial to the tibial tuberosity along with the depth determined by loss of resistance during needle insertion, was suitable for IO needle placement in Asian neonates. Secondary objectives were to determine the optimal depth of needle insertion and to assess the success rate using a drill-assisted device (DAD; Arrow^®^ EZ-IO^®^) compared to a manually inserted needle (MIN; Acufirm^®^ Sternal Puncture).

## Methods

This prospective study was conducted at Siriraj Hospital, the largest public quaternary care medical center in Bangkok, Thailand. The study protocol was approved by the Siriraj Institutional Review Board (COA no. Si 113/2023). Parental written consent was obtained prior to subject recruitment. Inclusion criteria comprised neonates of at least 23 weeks’ gestation who deceased within 28 days of life and were technically feasible for IO needle insertion within 24 h after death. The deceased infants who had passed away for more than 2 h were stored in a refrigerated cabinet at −2 to −4 °C in the Department of Pathology until ready for examination, without any immersion or injection of substances into the body. Exclusion criteria encompassed neonates with congenital leg anomalies, those whose families experienced severe depression following a neonatal death or were constrained by religious beliefs. Postnatal age at the time of death and duration between death and IO needle placement were recorded.

**Fig. 1:**
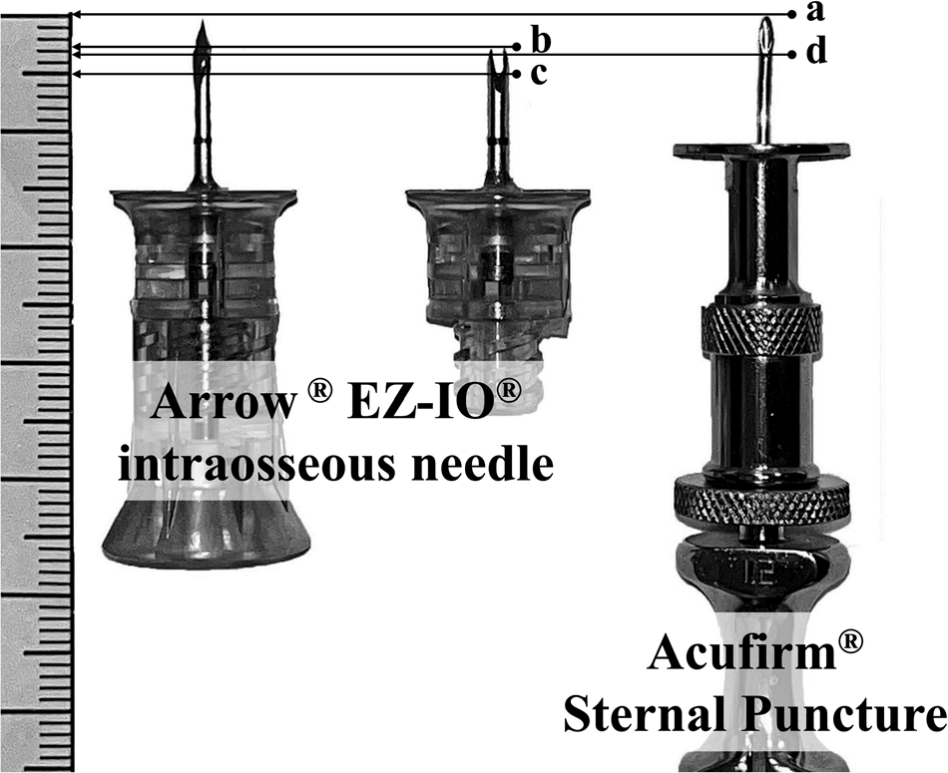
Features of the tip of the intraosseous needles. Black arrows indicate different positions of the needle tips as follows: (**a**) tip of Arrow^®^EZ-IO^®^ needle and Acufirm^®^ sternal needle, (**b**) tip of a diamond-shaped Arrow^®^EZ-IO^®^ needle, (**c**) proximal end of the diamond shaped Arrow^®^EZ-IO^®^ needle, and (**d**) proximal end of the Acufirm^®^ needle.

We investigated 2 types of IO needles: (1) the Arrow^®^ EZ-IO^®^ intraosseous needle (PD 15 G with a maximum depth of 15 mm, diameter 1.8 mm, Teleflex Medical, Dublin, Ireland) [9] which uses an electric drill while holding downward pressure for insertion, and (2) Acufirm^®^ Sternal Puncture (REF:1497-L size 1 with a maximum depth of 11.5 mm, diameter 1.2 mm, Acufirm, Germany) [10] which was manually inserted using a twisting motion. The insertions were alternated between the left and right legs according to the sequence of the neonatal cadavers, with only one type of IO needle utilized in each leg. The initial IO type was randomly selected and was followed by the alternate type. Currently, the Acufirm^®^ needle model 1497-L is suitable for reuse, while the EZ-IO^®^ needle is intended for single use and this practice was adopted in the study. Figure 1 illustrates the distinct features present at the tips of these needles. The EZ-IO^®^ needle has a diamond-shaped tip with a 2.5 mm length, accompanied by a stylet extending an additional 2.5 mm beyond the needle tip, designed to facilitate swift and gentle insertion. Conversely, the Acufirm^®^ needle features a bevel tip measuring 3 mm, with both the needle and stylet tips positioned at the same level, facilitating precise insertion, and reducing the likelihood of tip exposure beyond the medullary cavity.

All IO device insertions were solely performed by the principal investigator (CS) who is a neonatal fellow and had undergone training and instruction in the use of both types of needles on simulated tibial bones. The insertion site in neonatal cadavers aligned with the NRP guideline [3], targeting the flat area at 2 cm below and 1 cm medial to the tibial tuberosity. The needle was inserted perpendicular to the skin and penetrated through the periosteum, the bone cortex, reaching the medulla. The depth of needle penetration was determined based on the loss of resistance upon entering the marrow cavity. After stylet removal, a 5-mL syringe was employed to aspirate blood and flush the needle with 1 mL normal saline to ensure absence of subcutaneous tissue swelling before securing the needle with Nexcare™ Self-Adherent Coban Wrap, USA. The neonatal cadavers were immediately transferred to the Department of Radiology for a computed tomography (CT) scan.

**Fig. 2:**
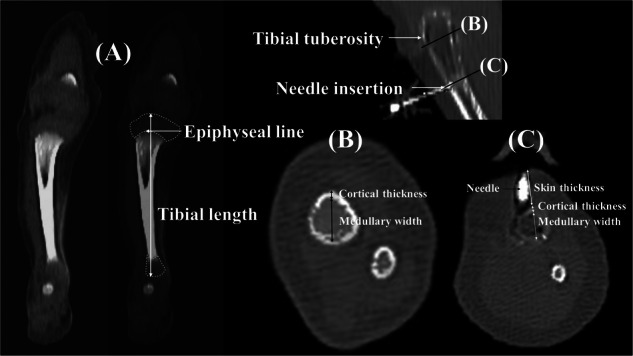
Radiological measurements from computed tomography scan images of the tibial bone. **A** Longitudinal view demonstrates measurement of tibial length. **B** Cross-sectional view measurements at the widest medullary cavity of the proximal tibial bones. **C** Cross-sectional view measurements at the intraosseous needle insertions.

Before CT scanning, 1 mL of contrast media (IOPAMIRO370^®^, ULTRAVIST370^®^, OMNIPAQUE350^®^) was injected via the inserted IO needles. Distribution of the contrast agent was assessed using Dual Energy CT (Revolution Apex, General Electric, USA). The data were reconstructed using the fast kV switching technique and were analyzed on a clinical SIPAC workstation by the pediatric radiologist (PP) who was blinded to the neonatal care of the subjects. Several anatomical measurements of the tibial bones were obtained from the CT scan, as depicted in Fig. 2. These measurements included tibial length from the longitudinal view, as well as measurements from the cross-sectional view at the widest medullary cavity of the proximal tibial bones and at the IO needle insertions, which encompass skin and cortical thickness, and medullary width. The depth of needle insertion was measured from the outermost aspect of the skin to the tip of the needle inside the leg. Successful insertion was confirmed by identification of the needle tip into the marrow cavity and observing the distribution of the radiopaque substance within the marrow cavity with or without contrast leakage (Supplementary Fig. 1).

### Statistical analysis

Based on the study by Fuchs et al. [11], the mean bone thickness was established at 1.19 mm with a standard deviation (SD) of 0.13 mm. These parameters were leveraged for sample size determination, factoring in a 95% confidence interval and an estimated margin of error of 0.06 (approximately 5% of the mean). A sample size of 19 subjects was required for the study.

Infant demographic characteristics are presented as numbers (percentage), mean ± SD, or median [25th percentile, 75th percentile; P25, P75] where appropriate. The appropriate needle insertion site at the proximal tibial bone, as evaluated from CT scan images, was determined by assessment at the widest width of the medulla and at the insertion site. The CT scans of the first 10 neonatal cadavers underwent repeated measurements for every parameter to analyze reliability, utilizing the intraclass correlation coefficients (ICC) statistical measure with a 95% confidence interval. Intra-rater reliability of successful insertion and contrast leakage was analyzed by Cohen’s kappa. All radiological measurements were reported descriptively and compared among the birthweight (BW) groups using ANOVA test. Comparisons of measurements between right and left legs were performed using paired *t*-test. The success rates of both types of IO insertion were compared using chi-square or Fisher exact test.

We proposed the appropriate minimum insertion depth based on the sum of the distance from the skin through the cortical thickness and the maximum insertion depth based on the sum of the distance from the skin through the cortex and medullary cavity. In order to apply the appropriate depth in clinical practice, we estimated the proportion of proper IO needle placements at each depth based on whether that depth fell between the minimum and maximum insertion depth for each infant.

Statistical analyses were conducted using IBM SPSS Statistics 20.0 software, with a predefined significance threshold set at *p* < 0.05.

## Results

From March 1st, 2023, to February 29th, 2024, a total of 23 neonatal deaths occurred in our hospital. However, four infants were excluded from the study due to the lack of timely parental consent. Thus, 19 neonatal cadavers (38 tibial bones) were included. Among them, 15 infants were of Thai nationality, while the remaining four were of Myanmar descent. The median gestational age at birth was 33.0 [28.0, 36.0] weeks, with a range spanning 24 to 40 weeks. Preterm infants (<37 weeks’ gestation) comprised the majority, accounting for 78.9% (15 cadavers). Birth weights ranged from 650 to 4100 g, with six cadavers classified as very-low birthweight infants (VLBW; birth weight <1500 g). The median postnatal age at the time of death was 4.0 [2.0, 8.0] days. Table 1 presents demographic characteristics of the subjects enrolled in the study. The median duration from death until IO needle placement was 8.0 [2.0, 12.0] hours, with nine cadavers having IO needles inserted within 2 h of death. Two infants who succumbed to hydrops fetalis exhibited only moderate edema, particularly in their legs, which appeared mildly edematous.

**Table 1:** Infant demographic characteristics (*N* = 19).

Gestational age	33.0 [28.0, 36.0]
Postnatal age at death (day)	4.0 [2.0, 8.0]
Male sex	13 (68.4)
Birth weight (g)	1680.0 [1110.0, 2440.0]
<1000	4 (21.1)
1000–1499	2 (10.5)
1500–2499	10 (52.6)
≥2500	3 (15.8)
Birth weight-for-gestational age status	
Appropriate-for-gestational age	11 (57.9)
Small-for-gestational age	5 (26.3)
Large-for-gestational age	3 (15.8)
Body length (cm)	40.0 [35.0, 45.0]
Duration from death to intraosseous needle insertion (hour)	8.0 [2.0, 12.0]
Principle causes of death	
Severe congenital anomalies^a^	5 (26.3)
Respiratory failure	4 (21.1)
Congenital critical heart disease	4 (21.1)
Necrotizing enterocolitis	3 (15.8)
Severe sepsis	1 (5.3)
Hydrops fetalis	2 (10.5)

Data are presented as median [25th percentile, 75th percentile] or number (%).

^a^2 infants with multiple congenital anomalies that included cerebral malformations and severe lung hypoplasia, 1 infant with trisomy 13, 1 infant with trisomy 14, and one with Down syndrome and transient myeloproliferative disorder.

Both types of IO needles were selected for use with each type being utilized for 19 initial insertions. Table 2 illustrates the performance of IO needle placements. The overall success rate of IO insertion was 86.8%, with comparable success rates observed between the EZ-IO^®^ and Acufirm^®^ needles (84.2% vs. 89.5%, respectively, *p* = 1.00). However, the success rate decreased to 66.7% in VLBW infants. Although the success rate was lower with EZ-IO^®^ needles compared to Acufirm^®^ needles, the difference was not statistically significant (50% vs. 83.3%, respectively; *p* = 0.55). Evidence of contrast leakage was detected in 6 of 38 insertions (15.8%) which was 41.7% in VLBW infants. Although the depth of needle insertion was similar between the two types of needles (0.94 ± 0.23 cm for EZ-IO^®^ vs. 0.90 ± 0.21 cm for Acufirm^®^, *p* = 0.64), EZ-IO^®^ needles exhibited a greater tendency towards contrast leakage compared to the Acufirm^®^ needles, although this difference did not reach statistical significance (21.1% vs. 10.5%, *p* = 0.66). The evidence of leakage was higher in VLBW infants (66.7% vs. 16.7% for EZ-IO^®^ and Acufirm^®^ needles respectively, *p* = 0.24). No fractures occurred in any tibial insertions.

**Table 2:** Successful placement of intraosseous needles (*N* = 38).

	Total	Type of catheter		*p
		EZ-IO^®^ needle	Acufirm^®^ needle	
All	(*N* = 38)	(*n* = 19)	(*n* = 19)	
Depth of needle penetration (cm)	0.92 ± 0.22	0.94 ± 0.23	0.90 ± 0.21	0.64
Successful insertion^a^	33 (86.8)	16 (84.2)	17 (89.5)	1.00
Contrast leakage	6 (15.8)	4 (21.1)	2 (10.5)	0.66
Birthweight < 1500 g	(*n* = 12)	(*n* = 6)	(*n* = 6)	
Depth of needle penetration (cm)	0.78 ± 0.20	0.78 ± 0.16	0.77 ± 0.25	0.90
Successful insertion^a^	8 (66.7)	3 (50.0)	5 (83.3)	0.55
Contrast leakage	5 (41.7)	4 (66.7)	1 (16.7)	0.24

*p < 0.05 is statistically significant.

^a^Identification of the intraosseous needle tip and observing the distribution of the radiopaque substance within the bone marrow cavity.

The ICC values of all radiological measurements exceeded 0.9 ((*p* < 0.001 for all) (Supplementary Table 1). Kappa coefficients for both successful insertion and contrast leakage were 1.00 (*p* < 0.001). Radiological measurements of the tibial bones, focusing on the proximal parts, are presented in Table 3. Notably, there were no discernible differences in measurements between the right and left tibial bones, as detailed in Supplementary Table 2. Skin thickness, tibial length, and cortical thickness showed increments with increasing BW groups. Measurement at the widest part of the medullary cavity revealed a mean of 0.37 ± 0.11 cm below the epiphyseal plate, with a mean cortical thickness of 0.21 ± 0.05 cm, and a mean medullary width of 0.47 ± 0.14 cm. The IO needle insertion site was approximately 1.46 ± 0.27 cm below the epiphyseal plate, which was similar across the BW groups, with a mean medullary cavity width of 0.21 ± 0.07 cm. The measurement from skin thickness through cortical thickness represents the minimum puncture depth, which was 0.62 ± 0.22 cm. Similarly, measuring from skin thickness through the cortex to the widest point of the medullary width represents the maximal distance to which the needle could be inserted, with a mean of 0.83 ± 0.25 cm. These minimum and maximum insertion depths increased in higher BW groups. The estimated rate of proper insertion based on appropriate depth and BW is presented in Supplementary Table 3. Only 1 insertion in a VLBW infant (8.3%) was proper when the depth exceeded 0.5 cm. For infants with a BW of 1500–2499 g, 12 insertions (60%) achieved proper insertion at a depth of 0.5 cm, and 7 insertions (35%) were proper at a depth of 0.75 cm. For those with a BW of 2500–3500 g, 4 insertions (100%) achieved proper insertion at a depth of 1 cm. For a BW ≥ 3500 g, there was only 1 infant with a BW of 4100 g who had appropriate minimum and maximum depths of 1.1 and 1.4 cm, respectively.

**Table 3:** Radiological characteristics of tibial bones (*N* = 38).

	All (*N* = 38)	Birthweight (g)				p*
		<1000 (*n* = 8)	1000–1499 (*n* = 4)	1500–2499 (*n* = 20)	≥2500 (*n* = 6)	
Tibial length (cm)	6.71 ± 1.64	4.64 ± 0.31	6.30 ± 0.55	6.89 ± 0.73	9.15 ± 1.78	<0.001*
Skin thickness (cm)	0.46 ± 0.19	0.28 ± 0.03	0.34 ± 0.04	0.48 ± 0.15	0.74 ± 0.16	<0.001*
Distance from epiphyseal growth line to tibial tuberosity (cm)	0.37 ± 0.11	0.29 ± 0.03	0.32 ± 0.05	0.37 ± 0.07	0.53 ± 0.17	<0.001*
Measurements at the widest medullary cavity of the proximal tibial bones						
Cortical thickness (cm)	0.21 ± 0.05	0.17 ± 0.05	0.19 ± 0.04	0.22 ± 0.05	0.27 ± 0.03	0.002*
Medullary cavity diameter (cm)	0.47 ± 0.14	0.44 ± 0.12	0.37 ± 0.02	0.48 ± 0.17	0.53 ± 0.04	0.33
Measurements at the intraosseous needle insertions						
Distance from epiphyseal growth line to the insertion site (cm)	1.46 ± 0.27	1.49 ± 0.16	1.41 ± 0.27	1.46 ± 0.31	1.47 ± 0.29	0.97
Cortical thickness (cm)	0.16 ± 0.04	0.13 ± 0.02	0.14 ± 0.03	0.15 ± 0.04	0.20 ± 0.02	0.01*
Medullary cavity diameter (cm)	0.21 ± 0.07	0.16 ± 0.03	0.24 ± 0.03	0.21 ± 0.08	0.26 ± 0.02	0.03*
Appropriated minimum insertion depth^a^ (cm)	0.62 ± 0.22	0.41 ± 0.05	0.48 ± 0.06	0.63 ± 0.18	0.94 ± 0.17	<0.001*
Appropriated maximum insertion depth^b^ (cm)	0.83 ± 0.25	0.56 ± 0.06	0.73 ± 0.04	0.84 ± 0.19	1.20 ± 0.17	<0.001*

Data are presented as mean ± standard deviation.

*P-values compare difference among the 4-birthweight groups*. p* < 0.05 is statistically significant.

^a^Distance from skin through cortical thickness.

^b^Distance from skin through cortex and medullary cavity.

## Discussion

The administration of medications or fluids is a critical step in neonatal resuscitation. Although only 0.5% of infants may require intensive resuscitation [1], these infants are typically the most critically ill and at risk of death or severe morbidities. In cases where umbilical venous catheterization is not feasible, IO needle insertion is widely accepted and effective. The NRP 8th edition recommends a single insertion site for needle placement, without specifying any differences between term and preterm infants [3]. However, various factors including gestational age, parental stature, and ethnicity can modify fetal growth. Previous studies showed that gestational age is a key predictor of fetal growth [12]. Tibial bones exhibit high rates of diaphyseal growth in utero [13]. Therefore, it is possible that tibial growth progresses with increasing gestational age, leading to changes in length and cortical thickness, which could potentially affect the depth and insertion site of the tibial bones. It is important to determine the appropriate IO insertion site and depth for each infant to ensure that the needle tip is positioned correctly without causing serious adverse events it is important to determine the appropriate IO insertion site and depth for each infant to ensure that the needle tip is positioned correctly without causing serious adverse events. Additionally, determining the appropriate depth of insertion using the sense of loss of resistance presents a challenge, particularly in infants with small tibial bones. Guidance regarding the insertion depth tailored to the size of each infant is therefore essential.

In our study, the typically recommended position for IO placement, which is 2 cm below the tibial tuberosity and 1–2 cm medially [3], was found to be at 1.46 ± 0.27 cm below the epiphyseal plate. Boon et al. [5] conducted a study involving the dissection of neonatal cadavers and IO needle insertion, 1 cm below the tibial tuberosity. They reported that the needle tip was approximately 10–15 mm away from the epiphyseal growth plate in this position. However, they did not measure the position at 2 cm below the tibial tuberosity due to difficulty in placement. Therefore, they suggested that the position 1 cm below the tibial tuberosity was suitable. We propound that this position may set the needle tip too close to the epiphyseal plate, posing a risk of injury, as evidenced by Schwindt et al. who reported the risk to be as high as 53% [14]. Therefore, we advocate for IO needle insertion at the recommended position of 2 cm below the tibial tuberosity, which is relatively safe and minimizes the risk of injury to the growth plate. Furthermore, the thin cortex observed at this position (mean: 0.16 cm) facilitates easier and faster insertion, requiring less pressure and reducing the likelihood of fracture. Cortical thickness measured in our cohort was similar to the study by Fuchs et al. who reported a thickness of 1.2 mm [11].

We observed an overall success rate of 86.8%, which was 66.7% in VLBW infants. This success rate was higher than the 40% to 61% range reported by Fuchs et al., dependent on the type of IO needles used [11]. During IO insertion, blood aspiration was observed from only 3 EZ-IO^®^ needles across 9.1% of successful insertions, with one of these occurring in a VLBW infant. While blood aspiration was possible, we cannot be certain about the rate of successful blood aspiration since our study involved neonatal cadavers that had been deceased for a median of 8.0 [2.0, 12.0] hours, during which time blood may have clotted. Hence, the absence of aspirated blood does not necessarily indicate IO malposition.

Reported procedural complications from leakage include hematoma or subcutaneous fat necrosis [7]. Among the 6 insertions (15.8%) that exhibited contrast leakage, one EZ-IO^®^ needle had its tip identified within the medullary cavity and displayed contrast filling. In real-world scenarios, instances like these may still permit some medication or infused volumes to flow into the systemic circulation. Therefore, we considered this as a successful placement. On the other hand, the remaining 3 EZ-IO^®^ and 2 Acufirm^®^ needles were not positioned within the medullary cavity and were thus deemed unsuccessful insertions. Overall, the EZ-IO^®^ needles had a higher, non-significant rate of contrast leakage compared to the Acufirm^®^ needles. This difference may be attributed to the tip design of the two needle types as outlined in the materials and methods section. The EZ-IO^®^ needle predisposes the needle to potentially protrude beyond the medullary cavity, which exhibited a mean size of 0.21 ± 0.07 cm across all birth weight groups. Specifically, in extremely low birth weight (ELBW; BW < 1000 g) infants, the mean medullary cavity size was measured at 0.16 ± 0.03 cm, increasing the likelihood of cortical penetration on the opposite side. A higher incidence of contrast leakage occurred in VLBW infants, particularly in the subgroup utilizing EZ-IO^®^ needles. The lack of statistical significance may be attributed to an insufficient sample size to adequately demonstrate a difference. Fuchs et al. [11], also found that IO insertions with a butterfly needle had a 2.4-fold significantly higher odds ratio of appropriate needle placement compared to EZ-IO^®^ needles. While EZ-IO^®^ needles can indeed be manually drilled into the bone, we opted to utilize the DAD, which is widely practiced. Therefore, our observations could potentially stem from either the type of needle tips or the insertion mechanism. However, since the depth of insertion was not different between the needle types (Table 2), we believe that it can be more likely attributed to the type of the needle tip. Additionally, since we did not observe any fractures with either the DAD or manual insertion using a twisted hand motion, we recommend the use of conventional manual needles, such as the Acufirm^®^, over the EZ-IO^®^ for IO insertion, particularly in ELBW infants.

Suominen et al. investigated the medullary diameter of neonatal tibial bones using x-ray imaging in full-term infants aged 1–28 days and reported a diameter of 7.7 ± 0.4 mm [6], which is considerably wider than the mean medullary cavity diameter of 2.6 mm observed in our neonatal cadavers weighing >2500 g. Several potential reasons may account for the discrepancy between the studies. First, Suominen et al. included 10 full-term infants in their study, whereas the three cadavers in our >2500 g group had gestational ages of 36, 38, and 39 weeks, with postnatal ages ranging from 2 to 7 days. Hence, both studies may have measured neonates with different demographic characteristics. Second, the techniques and position used for measurement differed. Suominen et al. measured the medullary diameter in the antero-posterior and lateral dimensions of x-rays taken 1 cm below the proximal end of the tibia due to technical difficulties in identifying the tibial tuberosity, while we utilized CT scanning images to measure the cross-sectional dimension at 2 cm below the tibial tuberosity. Although the measurement positions by Suominen et al. were also higher than in our study, they are close to our widest medullary cavity diameter of 5.3 mm in >2500 g infants. Last, potential differences may exist due to ethnic factors that contributed to variations in neonatal size. Fuchs et al. investigated formaldehyde-fixed stillbirth cadavers with gestational ages ranging from 26 to 43 weeks, utilizing spectral-CT examination to confirm successful insertion by identifying contrast media in the marrow cavity. The median diameter of the bone marrow cavity at the proximal tibia was reported as 4.0 mm [11], which was wider than our mean medullary diameter of 2.1 mm at the insertion position. However, it is important to note that Fuchs et al. documented the width of the medullary diameter at the proximal tibia, but the exact measurement level was not well defined. Therefore, their findings of the widest medullary cavity measurement of 4.7 mm may closely align with our study.

Based on our measurements, we recommend a minimal insertion depth of 0.62 ± 0.22 cm and a maximum distance of 0.83 ± 0.25 cm. However, the suggested depth should be selected based on the infant’s BW group. There may be concerns about exceeding the depth, especially with the DAD method. The EZ-IO^®^ needle for neonates has a maximum depth of 15 mm, marked by a 1 mm thick black line. The distance from the bottom edge of the black line to the base of the diamond-shaped tip is approximately 10 mm. Additionally, considering the 2.5 mm length of the diamond-shaped tip, inserting the needle until the bottom edge of the black line with the stylet removed would result in a needle tip depth in the marrow of approximately 5–7.5 mm (Fig. 1). Regarding the insertion depth for the Acufirm^®^ needle, it requires estimation based on the tip and total length of the needle. In the clinical setting, we propose convenient insertion depths for each birth weight group based on the estimated success rates presented in Supplementary Table 3. For VLBW infants, the insertion depth should be less than 0.5 cm or not exceeding the bottom edge of the black line on the EZ-IO^®^ needle. Infants weighing between 1500–2499 g should have an insertion depth between 0.5–0.75 cm, approximately at the level of the black line. For infants weighing 2500–3500 g, the insertion depth should be approximately 1 cm or extending a few millimeters beyond the upper edge of the black line to the base of the EZ-IO^®^ needle. For infants with a birth weight ≥3500 g, the needle should be inserted until the base. Hence, to enhance the likelihood of successful IO needle insertion, additional techniques may be necessary. These could include utilizing an angled rather than a perpendicular needle insertion or employing an ultrasound-guided placement [15]. These techniques can improve accuracy and reduce the risk of complications during the procedure [14]. Furthermore, we observed in our study that contrast media injected through IO needles could flow into the abdominal aorta and reach the heart, even in the absence of spontaneous circulation. This confirms that it is possible to flush a medication volume of just 1 mL through an IO needle to reach the heart.

This study aimed to investigate the appropriate insertion site and depth for IO needle placement in Asian infants, recognizing their typical smaller, anthropometric measurements. The positive attributes of our study are the apriori calculation of the sample size and the meticulously planned methodology. The assessment of the proper insertion site was conducted using standardized recommended positions, and needle insertion success was evaluated by confirming the presence of both the needle tip and contrast media in the marrow cavity, to ensure internal validity. Additionally, our study provides depth recommendations for IO needle insertion in Asian infants, categorized by BW groups. Despite the focus on only Asian neonatal cadavers, the cortical thickness and medullary cavity width was similar to previous studies conducted on infants of different ethnicities. Hence, the recommended insertion site and depth can be generalized and extrapolated for use in all infant populations without limitation to Asian neonates.

Several limitations of our study merit consideration. First, we did not exclude infants with hydrops fetalis recognizing that the IO route for the administration of medications and fluids during cardiovascular resuscitation, may be preferentially warranted over the chance of unsuccessful umbilical venous catheter placement in an edematous cord. Two infants with hydrops fetalis may have had thicker skin compared to normal infants, but their measured skin thickness ranged from 0.36 to 0.40 cm and was comparable to the other cadavers. Nevertheless, if IO needles need to be employed in infants with severe edema, deeper needle insertion may be necessary to compensate for the abnormal skin thickness at the insertion site. Second, the number of cadavers were relatively small to explore differences in the success rate between the types of IO needles. The success rate observed with the Acufirm^®^ needle in our study can be extrapolated to other needle brands that share the same bevel tip design. However, all the insertions were performed solely by the principal investigator of the study, which potentially enhanced the success rate beyond what might occur in real-world scenarios, where physicians performing IO insertions may not have extensive expertise. Lastly, we conducted our study on neonatal cadavers, which may undergo post-mortem changes that could potentially affect the measurement of skin thickness. However, the infants in this study were examined at a median duration of 8.0 [2.0, 12.0] hours after death and were stored in a cool environment to minimize post-mortem changes if the examination exceeded 2 h after death. Based on previous histological examination of skin changes after death [16], the epidermal and dermal layers mostly remained intact during this timeframe. Therefore, we feel that any potential effects on skin thickness measurements would be minimal. The clear definitions of insertion sites and depths from this study’s results afford generalizability of our findings and may increase confidence and improve the likelihood of successful insertions.

## Conclusions

The overall success rate of IO needle insertion in Asian neonates was 86.8%, which decreased to 66.7% in VLBW infants. We recommend the conventional IO insertion site in this population, located 2 cm below and 1–2 cm medially from the tibial tuberosity and advise the use of manually inserted needles over drill-assisted device placement, especially in ELBW infants, to avoid leakage. The correct IO insertion depth ranges from a minimum of 0.62 ± 0.22 cm to a maximum of 0.83 ± 0.25 cm, but the appropriate depth and safe medullary width should be determined based on birth weight which increases in infants with a higher birth weight.

## Supplementary information

Supplemental Table 1

Supplemental Table 2

Supplemental Table 3

Supplemental Fig. 1

## Data Availability

The datasets generated and/or analyzed during the current study are available from the corresponding author on reasonable request.
