# Supplementary material for: A prospective evaluation of tibial insertion sites for intraosseous needles to gain vascular access in Asian neonates
Source: J Perinatol. 2024 Jun 6;45(2):229–34. doi: 10.1038/s41372-024-02018-x (PMC11825351; doi:10.1038/s41372-024-02018-x)
Supplement: Supplementary file 1 — Supplemental Table 1 [file 41372_2024_2018_MOESM1_ESM.docx]

| **Supplemental Table 1 Intra-rater reliability of radiological measurements of the tibial bone (n =10)** | | | | |
| --- | --- | --- | --- | --- |
|  | | **Intraclass correlation**  **(95% confidence interval)** | ***p**** |  |
| Tibial length (cm) | | 1.00 (1.00, 1.00) | <0.001* |  |
| Skin thickness (cm) | | 1.00 (1.00, 1.00) | <0.001* |  |
| Distance from epiphyseal growth line to tibial tuberosity (cm) | | 0.98 (0.95, 0.99) | <0.001* |  |
| **Measurements at the widest medullary cavity of the proximal tibial bones** | | |  |  |
|  | Cortical thickness (cm) | 0.94 (0.85, 0.98) | <0.001* |  |
|  | Medullary cavity diameter (cm) | 0.91 (0.79, 0.96) | <0.001* |  |
| **Measurements at the intraosseous needle insertions** | | |  |  |
|  | Distance from epiphyseal growth line to insertion point (cm) | 0.97 (0.93, 0.99) | <0.001* |  |
|  | Cortical thickness (cm) | 0.91 (0.79, 0.96) | <0.001* |  |
|  | Medullary cavity diameter (cm) | 0.91 (0.79, 0.96) | <0.001* |  |
| **p* <0.05 is statistically significant. | | | |  |
