# Supplementary material for: A prospective evaluation of tibial insertion sites for intraosseous needles to gain vascular access in Asian neonates
Source: J Perinatol. 2024 Jun 6;45(2):229–34. doi: 10.1038/s41372-024-02018-x (PMC11825351; doi:10.1038/s41372-024-02018-x)
Supplement: Supplementary file 2 — Supplemental Table 2 [file 41372_2024_2018_MOESM2_ESM.docx]

| **Supplemental Table 2 Comparison of radiological measurements between each side of the tibial bones** | | | | | | | | | | | | | | | | |  |  |
| --- | --- | --- | --- | --- | --- | --- | --- | --- | --- | --- | --- | --- | --- | --- | --- | --- | --- | --- |
|  | | **All**  **(N=19)** | | | **Birthweight (g)** | | | | | | | | | | | |  |  |
|  |  |  |  |  | **<1000**  **(n=4)** | | | **1000-1499**  **(n=2)** | | | **1500-2499**  **(n=10)** | | | **≥2500**  **(n=3)** | | | |  |
|  |  | **Left** | **Right** | ***p**** | **Left** | **Right** | ***p**** | **Left** | **Right** | ***p**** | **Left** | **Right** | ***p**** | **Left** | **Right** | ***p**** | |  |
| Tibial length (cm) | | 6.71 ± 1.66 | 6.71 ± 1.66 | 0.88 | 4.65 ±0.34 | 4.64 ± 0.33 | 0.32 | 6.31 ± 0.67 | 6.30 ± 0.67 | -** | 6.89 ± 0.75 | 6.89 ± 0.76 | 0.54 | 9.14 ± 2.01 | 9.17 ± 1.96 | 0.49 | |  |
| Skin thickness (cm) | | 0.46 ± 0.19 | 0.46 ± 0.20 | 0.57 | 0.28 ± 0.05 | 0.28 ± 0.02 | 0.72 | 0.35 ± 0.04 | 0.33 ± 0.04 | -** | 0.48 ± 0.16 | 0.47 ± 0.16 | 0.10 | 0.72 ± 0.15 | 0.75 ± 0.19 | 0.41 | | |
| Distance from epiphyseal growth line to tibial tuberosity (cm) | | 0.37 ± 0.12 | 0.37 ± 0.10 | 0.96 | 0.30 ± 0.04 | 0.29 ± 0.02 | 0.67 | 0.32 ± 0.08 | 0.32 ± 0.03 | 0.91 | 0.36 ± 0.07 | 0.38 ± 0.06 | 0.02* | 0.56 ± 0.20 | 0.49 ± 0.18 | 0.11 | | |
| \| **Measurements at the widest medullary cavity of the proximal tibial bones** \| \| --- \| | | | | | | | | | | | | | | | | |  |  |
|  | Cortical thickness (cm) | 0.21 ± 0.06 | 0.21 ± 0.06 | 0.69 | 0.17 ± 0.05 | 0.17 ± 0.05 | 0.79 | 0.19 ± 0.02 | 0.19 ± 0.06 | 1.00 | 0.22 ± 0.05 | 0.21 ± 0.05 | 0.72 | 0.27 ± 0.03 | 0.27 ± 0.04 | 1.00 | | |
|  | Medullary cavity diameter (cm) | 0.47 ± 0.14 | 0.47 ± 0.14 | 0.33 | 0.44 ± 0.13 | 0.45 ± 0.14 | 0.55 | 0.36 ± 0.01 | 0.39 ± 0.01 | 0.26 | 0.48 ± 0.17 | 0.48 ± 0.18 | 0.94 | 0.52 ± 0.05 | 0.54 ± 0.05 | 0.63 | | |
| **Measurements at the intraosseous needle insertions** | | | | | | | | | | | | | | | | |  |  |
|  | Distance from epiphyseal growth line to insertion point (cm) | 1.47 ± 0.26 | 1.44 ± 0.29 | 0.52 | 1.52 ± 0.11 | 1.46 ± 0.22 | 0.73 | 1.37 ± 0.04 | 1.44 ± 0.45 | 0.85 | 1.45 ± 0.31 | 1.46 ± 0.33 | 0.87 | 1.57 ± 0.35 | 1.37 ± 0.23 | 0.18 | |  |
|  | Cortical thickness (cm) | 0.16 ± 0.04 | 0.15 ±0.04 | 0.70 | 0.13 ± 0.03 | 0.13 ± 0.02 | 1.00 | 0.14 ± 0.02 | 0.15 ± 0.04 | 0.50 | 0.16 ± 0.05 | 0.15 ± 0.04 | 0.41 | 0.20 ± 0.03 | 0.20 ± 0.02 | 0.42 | |  |
|  | Medullary cavity diameter (cm) | 0.21 ± 0.07 | 0.21 ± 0.07 | 0.62 | 0.16 ± 0.03 | 0.16 ± 0.03 | 1.00 | 0.24 ± 0.02 | 0.25 ± 0.04 | 0.50 | 0.21 ± 0.08 | 0.21 ± 0.09 | 0.66 | 0.26 ± 0.02 | 0.26 ± 0.02 | 0.18 | |  |
|  | Data are presented as mean ± standard deviation.  *P-values compare differences between the left and right tibial measurements. *p* <0.05 is statistically significant.  ** Statistical analysis not applicable due to zero standard error | | | | | | | | | | | | | | | |  |  |
