# Supplementary material for: A prospective evaluation of tibial insertion sites for intraosseous needles to gain vascular access in Asian neonates
Source: J Perinatol. 2024 Jun 6;45(2):229–34. doi: 10.1038/s41372-024-02018-x (PMC11825351; doi:10.1038/s41372-024-02018-x)
Supplement: Supplementary file 3 — Supplemental Table 3 [file 41372_2024_2018_MOESM3_ESM.docx]

| **Supplemental Table 3 Estimated proportion of proper placement of intraosseous needles based on insertion depth and birthweight (N=38)** | | | | | |
| --- | --- | --- | --- | --- | --- |
| **Birthweight group (g)** | **Depth of needle insertion (cm)** | | | |  |
|  | **0.5** | **0.75** | **1.0** | **>1.0** |  |
| <1500 (n= 12) | 12 (100.0) | 1 (8.3) | 0 (0.0) | 0 (0.0) |  |
| 1500-2499 (n=20) | 12(60) | 7 (35.0) | 5 (25.0) | 2 (10.0) |  |
| 2500-3499 (n=4) | 0 (0.0) | 0 (0) | 4 (100.0) | 2 (50.0) |  |
| ≥3500 (n=2) | 0 (0.0) | 0 (0.0) | 0 (0.0) | 2 (100.0) |  |
| Data presented as number (percentage) | | | | | |
