# Supplementary figures and images for: A prospective evaluation of tibial insertion sites for intraosseous needles to gain vascular access in Asian neonates
Source: J Perinatol. 2024 Jun 6;45(2):229–34. doi: 10.1038/s41372-024-02018-x (PMC11825351; doi:10.1038/s41372-024-02018-x)

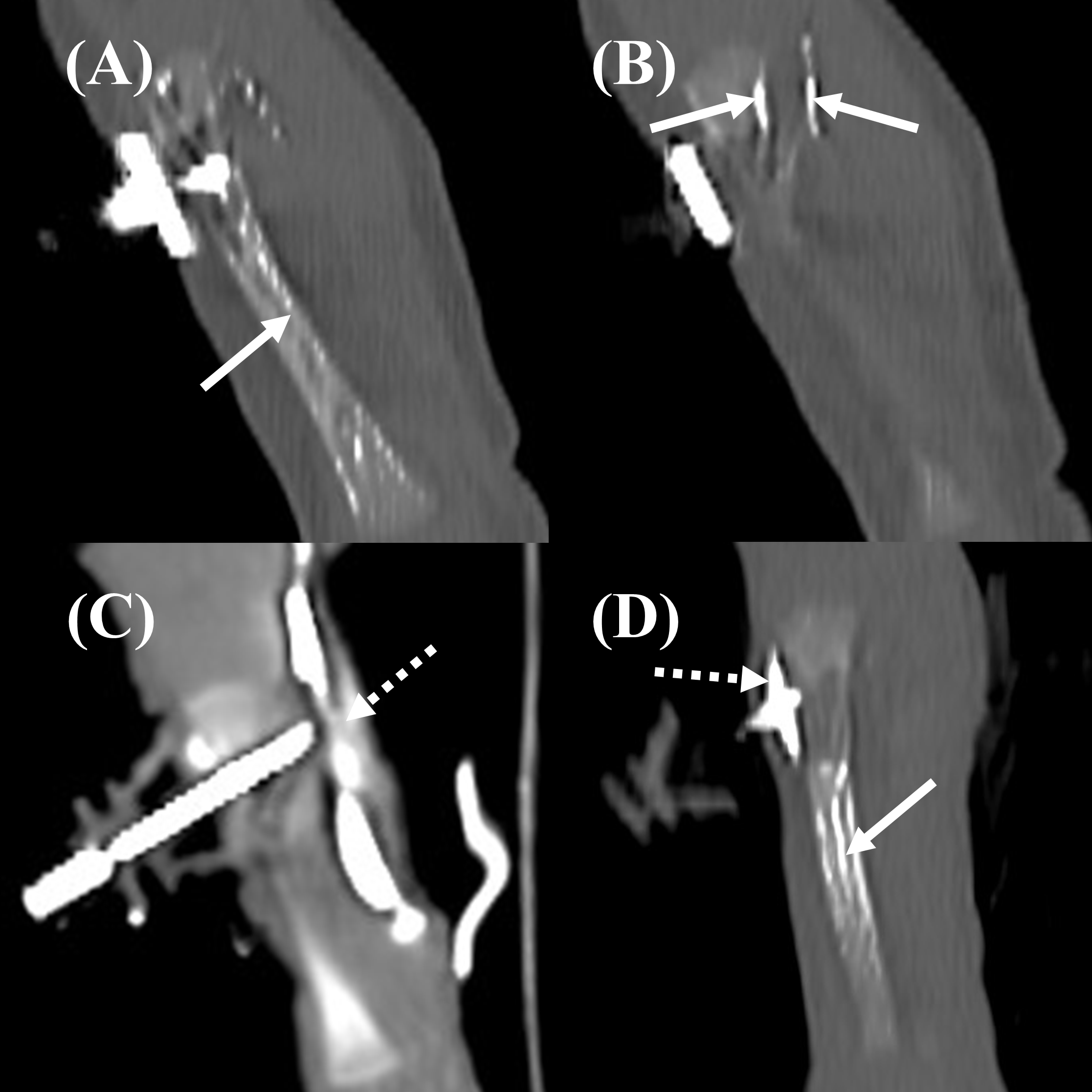

Supplement: Supplementary file 4 — Supplemental Fig. 1 [file 41372_2024_2018_MOESM4_ESM.tif]
